# Mitochondrial Changes in Ageing *Caenorhabditis elegans* – What Do We Learn from Superoxide Dismutase Knockouts?

**DOI:** 10.1371/journal.pone.0019444

**Published:** 2011-05-18

**Authors:** Jan Gruber, Li Fang Ng, Sheng Fong, Yee Ting Wong, Soon Ann Koh, Ce-Belle Chen, Guanghou Shui, Wei Fun Cheong, Sebastian Schaffer, Markus R. Wenk, Barry Halliwell

**Affiliations:** 1 Department of Biochemistry, Yong Loo Lin School of Medicine, National University of Singapore, Singapore, Singapore; 2 Department of Biological Sciences, Faculty of Science, National University of Singapore, Singapore, Singapore; University of South Florida College of Medicine, United States of America

## Abstract

One of the most popular damage accumulation theories of ageing is the mitochondrial free radical theory of ageing (mFRTA). The mFRTA proposes that ageing is due to the accumulation of unrepaired oxidative damage, in particular damage to mitochondrial DNA (mtDNA). Within the mFRTA, the “vicious cycle” theory further proposes that reactive oxygen species (ROS) promote mtDNA mutations, which then lead to a further increase in ROS production. Recently, data have been published on *Caenorhabditis elegans* mutants deficient in one or both forms of mitochondrial superoxide dismutase (SOD). Surprisingly, even double mutants, lacking both mitochondrial forms of SOD, show no reduction in lifespan. This has been interpreted as evidence against the mFRTA because it is assumed that these mutants suffer from significantly elevated oxidative damage to their mitochondria. Here, using a novel mtDNA damage assay in conjunction with related, well established damage and metabolic markers, we first investigate the age-dependent mitochondrial decline in a cohort of ageing wild-type nematodes, in particular testing the plausibility of the “vicious cycle” theory. We then apply the methods and insights gained from this investigation to a mutant strain for *C. elegans* that lacks both forms of mitochondrial SOD. While we show a clear age-dependent, linear increase in oxidative damage in WT nematodes, we find no evidence for autocatalytic damage amplification as proposed by the “vicious cycle” theory. Comparing the SOD mutants with wild-type animals, we further show that oxidative damage levels in the mtDNA of SOD mutants are not significantly different from those in wild-type animals, i.e. even the total loss of mitochondrial SOD did not significantly increase oxidative damage to mtDNA. Possible reasons for this unexpected result and some implications for the mFRTA are discussed.

## Introduction

It is often suggested that ageing is a consequence of damage accumulation [1], [2]. However, it is still unclear exactly how damage accumulation might lead to the time-dependent exponential increase in the risk of dying that is a defining characteristic of the ageing process [3]. While various types of damage do indeed accumulate with age, what types of damage affecting which biomolecular targets, if any, determine the rate of ageing remains unclear.

One of the most popular damage accumulation theories of ageing has been the mitochondrial free radical theory of ageing (mFRTA), proposing that ageing is due to the accumulation of partially unrepaired oxidative damage, in particular to mitochondrial DNA (mtDNA), resulting in mtDNA mutations [4]–[7]. If damage accumulation were to explain exponential mortality dynamics, we *might* (but do not have to, see: [8]) expect damage to be autocatalytic, which would imply exponential damage accumulation, in turn explaining the observed exponential increase in the risk of dying. In the context of the mFRTA, such an autocatalytic mechanism has been proposed. The “vicious cycle” variant of the mFRTA proposes that reactive oxygen species (ROS) cause mtDNA mutations which, by destabilising the mitochondrial electron transport chain (ETC), further increase ROS production [5], [9], [10].

However, while oxidative damage to DNA, proteins and lipids indeed occurs commonly *in vivo*, most such damage is efficiently repaired and, consequently, steady state levels of such damage in healthy individuals are usually low [11]. Evidence for the mFRTA in general is at best equivocal and it is far from clear whether oxidative damage plays a causative role in ageing [12]–[14]. In particular, data regarding the damage and mutation burden in mtDNA are sparse, in part due to technical limitations [11]–[13], [15].

Due to its relative simplicity, ease of genetic manipulation and short lifespan, the nematode worm *Caenorhabditis elegans (C. elegans)* is an attractive model to explore some of the open questions regarding the role of damage accumulation in ageing [16]. Since the first single-gene ageing mutation (*age-1*) was described in *C. elegans* [17], a large amount of data has been generated regarding ageing and its genetic determinants in nematodes [14], [18].

Ageing cohort studies have provided evidence for early and rapid decline in parameters of energy metabolism as well as structural deterioration in mitochondria with age in *C. elegans* [14], [19]–[21]. Furthermore, markers of global oxidative damage, including lipofuscin and total protein carbonyl content, a marker of oxidatively damaged protein, accumulate with age; this accumulation is more rapid in short-lived but slower in long-lived mutant strains [19], [22]–[25]. While these data support the notion that oxidative damage is *correlated* with metabolic and functional decline during ageing, it is hard to infer direct association let alone causality from such observations.

To overcome some of the limitations inherent in correlative ageing cohort studies, mutant strains have been generated by perturbing antioxidant systems with the aim of modulating oxidative damage. Superoxide dismutase (SOD) is one important antioxidant enzyme, responsible for the conversion (dismutation) of superoxide into hydrogen peroxide and oxygen, the first step of superoxide detoxification [11], [26]. *C. elegans* has two isoforms of mitochondrial SOD (encoded by *sod-2* and *sod-3*), two cytosolic SODs (*sod-1* and *sod-5*) and one extracellular SOD (*sod-4*) [27]. Recently, data have been published on mutants deficient in one or both forms of mitochondrial SOD. Surprisingly, in contrast to observations in yeast, flies and mice, it appears that in *C. elegans* neither deletion of *sod-2* nor *sod-3* decreases survival. Even *sod-2/sod-3* double mutants, entirely deficient in mitochondrial SOD, show no reduction in lifespan. In fact, some authors have reported a significant *extension* of lifespan for *sod-2* and *sod-2/sod-3* double mutants [28]–[31]. This lack of detrimental effects of mitochondrial SOD depletion on lifespan was observed despite clearly increased susceptibility to exogenous ROS and increased protein oxidation, as judged by elevated protein carbonyl levels [28]–[30]. These observations have been interpreted as strong evidence against a central role of ROS in determining lifespan and ageing rate, at least in nematodes [12], [24], [28], [32], [33]. This sentiment was further supported by fascinating evidence suggesting that elevated mitochondrial superoxide levels may actually be required for lifespan extension in some ETC mutant strains [34].

However, it is worth remembering that even supposedly simple model organisms are still immensely complex biological regulatory systems and often do not respond to perturbations in the way expected. Since mitochondrial SOD is entirely absent in the *sod-2/sod-3* mutants throughout life, including during development, it is possible that they are fundamentally different from wild-type (WT) animals in terms of mitochondrial function and ROS production.

To test for the possibility of compensation, changes in gene expression patterns have been examined as functions of overexpression and deletion of various SOD genes. Some compensatory upregulations of antioxidant and stress response genes, including isoforms of glutathione S-transferase, have indeed been reported in response to deletion of *sod-2* and *sod-3* [27], [30]. However, not all homeodynamic responses in living systems are mediated by changes in gene expression levels. For instance, overexpression of antioxidant enzymes can lead to the superficially surprising result of actually *elevating* ROS-mediated damage *in vivo*, as in the case of SOD overexpression in mammalian cells, flies and mice (reviewed in: [35], [36]). The underlying mechanisms for this observation are complex, likely involving interactions between elements of the antioxidant systems and the ETC, but do not necessarily require changes in gene expression [35]–[38]. This link between ROS defence systems and the ETC is particularly relevant here because, in *C. elegans* reduction of ETC activity is commonly associated with extended lifespan, although the mechanism of this extension remains to be elucidated [14], [39].

It may be dangerous to assume that SOD mutants suffer from elevated steady state levels of oxidative damage to mitochondria and mtDNA without strong experimental support. Interestingly, there is one report of unchanged levels in aconitase activity in the mitochondria of *sod-2* and *sod-3* mutants, arguing against dramatically increased levels of oxidative stress in these animals [28]. To really answer this question, direct measurements of oxidative damage to mitochondrial macromolecules are required. However, measuring ROS-mediated mitochondrial damage is challenging, especially given the limited biological material available from nematodes. To date no methods for the determination of mtDNA specific damage in *C. elegans* or data regarding age-dependent mtDNA damage have been reported. This is a major challenge in making sense of the longevity patterns observed in ageing mutants [12]. We address some of these questions by quantifying age-dependent damage accumulation, including damage to mtDNA in ageing WT nematodes and by comparing damage levels between WT and *sod-2/sod-3* double mutants.

## Results

The purpose of the first part of this study is to follow a single cohort of ageing nematodes while simultaneously determining and correlating indicators of functional, metabolic and mitochondrial decline as well as of oxidative stress and damage. For this purpose, we developed three novel mitochondrial assays: one for single worm mtDNA copy number determination, a second for sequence-specific mtDNA damage and finally a lipidomics method for the characterisation of mitochondrial cardiolipin species (CL). While some of the parameters reported here have been assayed in ageing *C. elegans* previously, determining multiple closely related parameters in parallel and in the same cohort allows us to test the plausibility of various assumptions and predictions of the mFRTA and of the “vicious cycle” theory. In the second part of this study, we applied the assays and insights gained from the ageing cohort study to obtain new insights into the metabolic state of a mitochondrial *sod-2/sod-3* double knockout mutant strain (GA480). Together with a simple computer model, aimed at explaining the consequences of SOD modulation in the mitochondrial compartment, we then use these insights to further explore the question whether the lack of lifespan reduction in GA480 really represents a strong challenge to the notion that ROS might contribute to the determination of lifespan in *C. elegans*.

### Functional decline in ageing cohort

**Figure 1:**
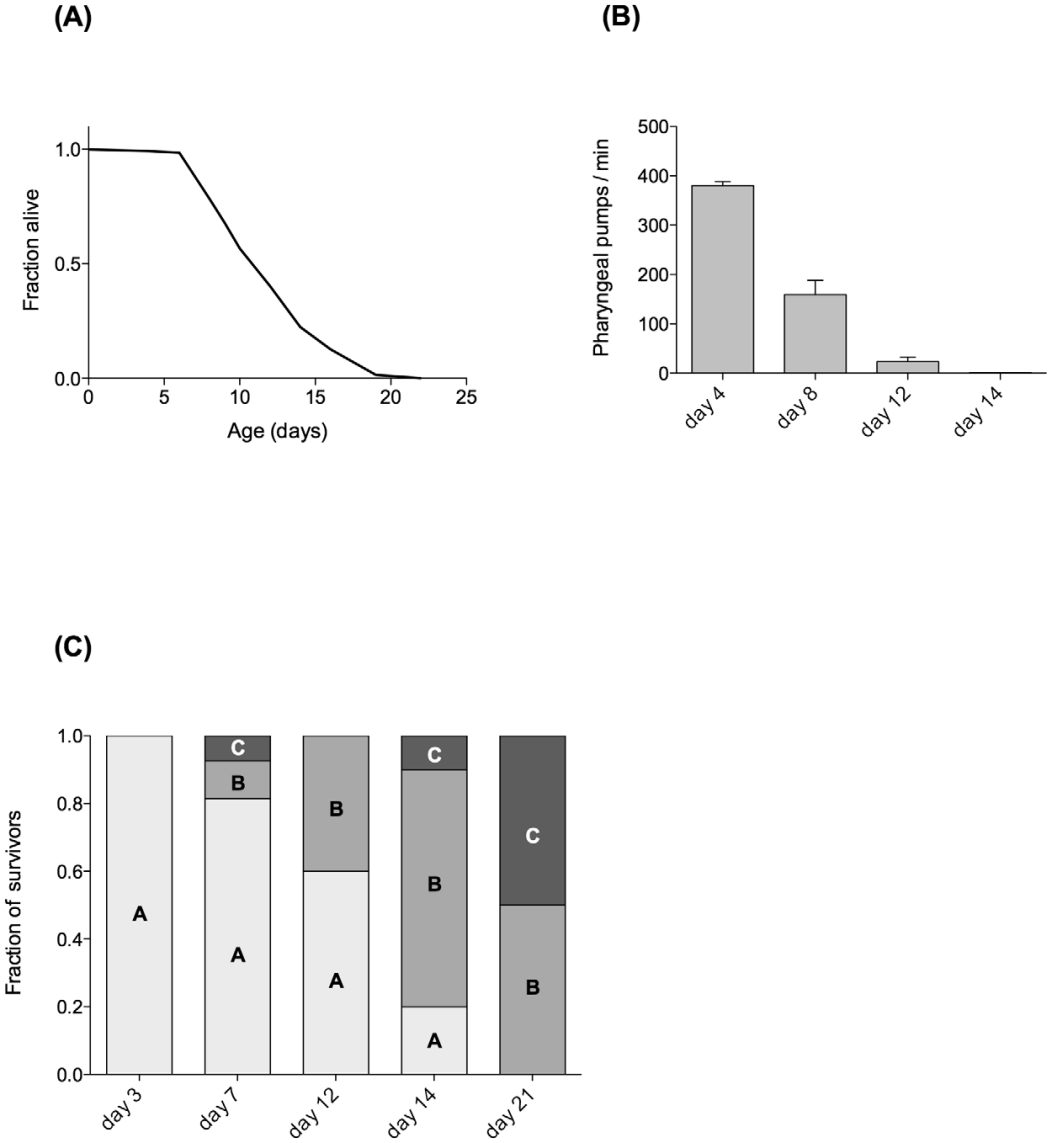
Age-dependent survival and functional decline in a typical JK1107 (*glp-1*) *C. elegans* cohort. Nematodes are maintained at the restrictive temperature of 25.5°C. (**A**) Survival curve. Mean lifespan 12.3 days (n = 134). (**B**) Functional decline as judged by reduction in pharyngeal pumping rate (mean ± SEM) with age. Worms exhibiting irregular or sporadic pumping were excluded. (**C**) Motility phenotype of surviving worms at different ages. Scoring was carried out according to [40]. Briefly, class “A” worms move vigorously, leaving sinusoidal tracks. Class “B” worms move only in response to touch or appear uncoordinated in locomotion. Class “C” worms cannot move their body but exhibit head and/or tail movements in response to touch.

The data presented here are from a single large cohort of JK1107 *C. elegans* although all observations are representative of at least two independent cohorts. To judge the functional state at each ageing time point and as a means of quality control, we determined survival, pharyngeal pumping and motility phenotype (a measure of health span [40], [41]) in all our cohorts. Figure 1 illustrates the impact of ageing on indicators of functional health. Mean lifespan was 12.3 days while maximum lifespan, defined as the average of the longest surviving 10% of the population, was 19 days (Figure 1A). These values are typical for the JK1107 strain at its restrictive temperature of 25.5°C [42], [43]. Pharyngeal pumping rates (Figure 1B) and motility phenotype (Figure 1C) are indicators of healthy functioning of the muscular and nervous systems. Both showed rapid age-dependent decline with few individuals able to maintain rhythmic pharyngeal pumping beyond day 12 (Figure 1B) and only 60% of animals retaining the highest score (“A”, most healthy) on day 12 (Figure 1C).

### Age-dependent decline in energy metabolism, mitochondrial content and function

**Figure 2:**
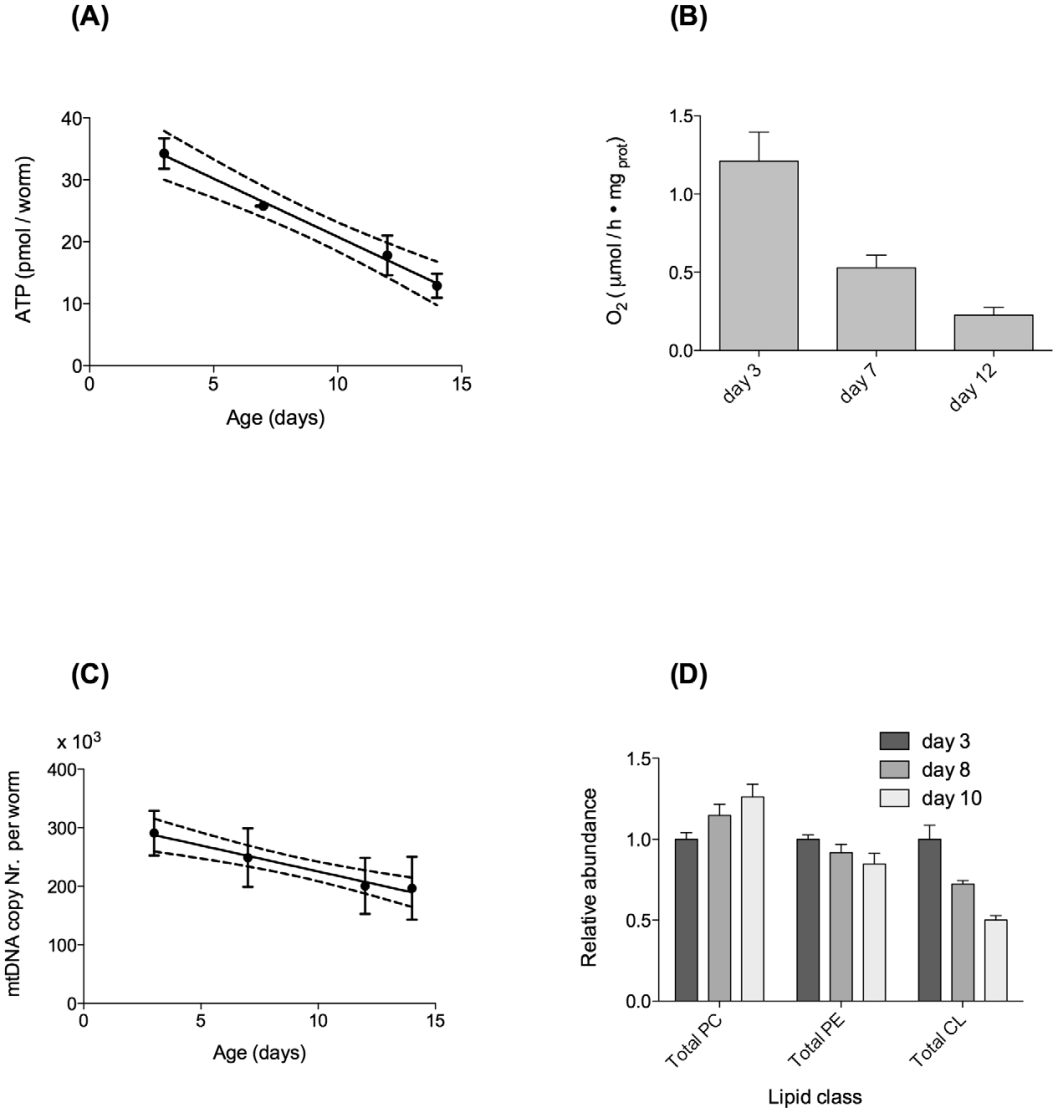
Metabolic parameters decline linearly with age in a typical JK1107 cohort. (**A**) Age-dependent decline in ATP content and linear regression. Best linear fit (R squared 0.86) and 95% confidence band. Slope statistically significantly different from zero (P<0.0001). (**B**) Age-dependent decline in oxygen consumption, normalised to worm protein. Decline in oxygen consumption was statistically significant (P = 0.003, ANOVA with Tukey's post-test). (**C**) Age-dependent decline in mtDNA copy number per worm and linear regression. Best linear fit (R squared 0.98) and 95% confidence bands. Slope statistically significantly different from zero (P<0.01). (**D**) Age-dependent loss of the mitochondrial membrane lipid cardiolipin in *C. elegans* total lipid extract (n = 3 independent extractions for each time point). Total CL content clearly declines with age (P<0.005). Lipid content is expressed as relative abundance of phosphatidylcholines (PC), phosphatidylethanolamines (PE) and Cardiolipin (CL), normalised to PC content of young worms.

In parallel with our observations on age-dependent functional decline, we determined markers of metabolic and mitochondrial function in JK1107 *C. elegans*. Figure 2A illustrates that the observed functional decline was associated with a significant, linear decline in adenosine triphosphate (ATP) levels with age. ATP levels decline by 5.7% (of reference levels on day 3) per day or 62% in total between days 3 and 14. This age-dependent decrease in ATP is reflected in worm metabolism, as determined by oxygen consumption (Figure 2B). While oxygen consumption at day 14 could not be reliably determined due to the low number of surviving worms, we found that oxygen consumption declined significantly with age between days 3 and 12 (Figure 2B). Consistent with some [19], [20], [44], [45] but not all [21], [46] previous studies, this decline was better fitted by an exponential (R squared = 0.98) than a linear decline (R squared = 0.79). However, with only three time-points, it is difficult to judge the significance of this observation. To determine if the age-dependent decline in metabolic function might be associated with loss of mitochondrial number, we determined mtDNA copy number in individual worms as a function of age. In contrast to one previous study [46], we found a statistically significant linear decline in mtDNA between days 3 and 14 (Figure 2C). However, this decline was relatively slight with copy numbers in old worms (day 14) on average about 27% lower compared to young worms (day 3). This moderate but significant decline in mtDNA copy number was accompanied by a statistically significant decline in the total worm content of the diphosphatidylglycerol lipid cardiolipin (CL). CL is mainly found in the inner mitochondrial membrane where it regulates mitochondrial function. CL is highly sensitive to damage by ROS due to its high content of polyunsaturated fatty acids and its location near sites of ROS production in the inner mitochondrial membrane. We found that total CL content was approximately 50% lower in older (day 10) animals compared to young (day 3) animals (Figure 2D). Together, these data show that a significant age-dependent decline in energy metabolism in ageing *C. elegans* may be related to a significant decline in mitochondrial number and function.

### Successful ageing is associated with preserved mtDNA copy number

**Figure 3:**
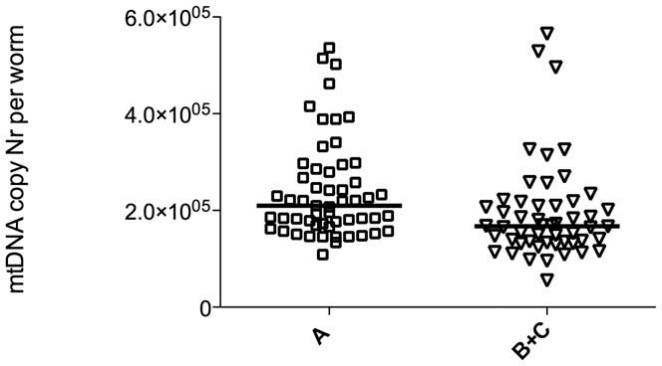
Successfully ageing (class “A”) old worms have higher mtDNA copy number than animals showing motility defects. Comparison of mtDNA copy number in JK1107 worms from the same cohort (identical genotype and environment) scored at the same age (day 12) but found to be in different motility classes. Graph shows scatter and median of individual worm copy number because data are not normally distributed. Class “A” worms have statistically significantly higher mtDNA copy number (median 210,000 vs. 170,000 copies) than class “B” and “C” animals (P<0.001, Mann-Whitney test). The n-numbers for class “A” and “B”/”C” were 55 and 52 respectively, although we typically found very few class “C” worms on day 12.

Given the evidence for a significant yet modest decline in mtDNA copy number with age, we sought a way to answer the question whether mtDNA copy number matters functionally to healthy ageing. To address this issue, we took advantage of the fact that our mtDNA copy number assay is performed in *individual* animals. This allows us to ask the question if worms that age more successfully, as judged by their motility phenotype, have on average higher (more preserved) mtDNA copy number than worms that age less well. For this experiment we selected approximately 50 animals each of class “A” and class “B” or “C” out of a population of 12-day-old animals. We then individually determined their mtDNA copy number. While there was significant overlap between the two groups, we found that worms that aged more successfully (remained in motility class “A”) had statistically significantly higher average mtDNA copy number than animals that were scored as class “B” or “C” on day 12 of life (Figure 3). Assuming that both groups started out with an average copy number for young animals (290,000 mtDNA copies per worm), mtDNA copy number in more and less successfully ageing individuals in this cohort declined on average by 28% and 41%, respectively. This illustrates that, while mtDNA copy number declined in both groups, more successful ageing appears to correlate with a slower decline in mtDNA copy number.

### Age-dependent increase in ROS and oxidative damage

**Figure 4:**
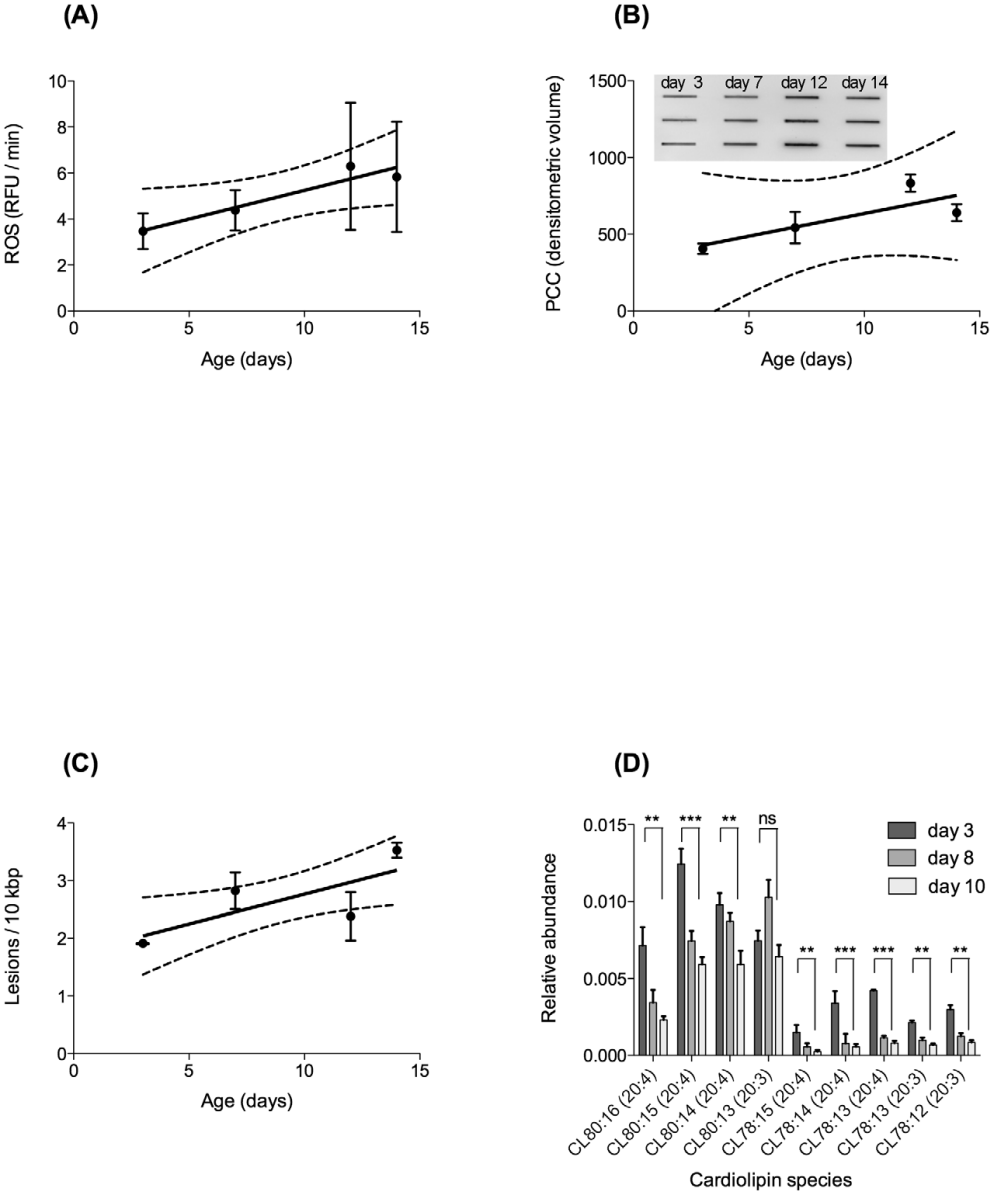
Age-dependent changes in parameters of ROS production and oxidative damage. (**A**) Generalised ROS production as evaluated using whole worm DCF-DA assay in intact animals. ROS production increases linearly with age and the linear regression of n = 9 replicates (R squared 0.91) has a positive slope, significantly different from zero (P<0.05). (**B**) Age-dependent protein carbonyl content (PCC), normalised to worm protein, slot blot and linear regression. Linear regression (R squared 0.66) cannot be used to reject zero slope (P = 0.19). However, protein carbonyl content in old animals is significantly higher than in young animals (P = 0.0003, ANOVA with Tukey's post-test). (**C**) Age-dependent increase in oxidative damage to mtDNA as measured by our qRT-PCR assay and expressed as lesions per bp of mtDNA. Oxidative mtDNA damage increases linearly with age (P = 0.006, ANOVA with Tukey's post-test). (**D**) Age-dependent change in the degree of saturation of the mitochondrial specific lipid cardiolipin (CL). Note that the CL species of 80∶13 has a combination of fatty acids of 3×20∶3 and 1×20∶4; CL 80∶14 = 2×20∶3 and 2×20∶4; CL 80∶15 = 1×20∶3 and 3×20∶4; CL 80∶16 = 4×20∶4. While the abundance of most species of CL declines with age, the more unsaturated species (with a higher number of double bonds) decline more dramatically with age than the corresponding, more saturated forms (comparison day 3 vs. day 10. ns: not significant, *: P<0.05, **: P<0.01, ***: P<0.01).

According to the free radical theory of ageing, age-dependent functional decline should be associated with a significant increase in oxidative damage, in particular to mtDNA. To test this prediction, we determined age-dependent changes in ROS production and damage accumulation using four different assays. Dichlorofluoresceine-diacetate (DCF-DA) is a popular fluorescence-based probe, commonly utilised to detect generalised ROS production. DCF-DA is first deacetylated by endogenous esterases to dichlorofluorescein (DCFH), which can react with several ROS to form the fluorophore DCF (reviewed in: [11]). While there is significant controversy regarding the actual ROS responsible for DCF-DA fluorescence, when used in whole *C. elegans*, it may be a useful indicator of global ROS flux in intact animals [47], [48]. Using the average rate of DCF-DA fluorescence in whole worms as proxy for ROS flux, we found a statistically significant, linear increase in ROS production with age (Figure 4A). However, the increase in ROS production rate was small, never even reaching levels twice as high as baseline.

We next determined three different indicators of oxidative damage. Total protein carbonyl content is a common marker for global oxidative protein damage [11], [49]. In agreement with previous work [19], we found a significant increase in total protein carbonyl content with age with maximal levels (day 12) approximately double those of young animals (Figure 4B). This increase appeared to be linear rather than exponentially accelerating, although variability in the data was high. Protein carbonyl content in whole worm lysate combines contributions originating from the cytoplasmic, extracellular and mitochondrial compartments. To investigate more specifically how oxidative damage burden in mitochondria changes with age, we developed a novel sequence-specific mtDNA damage assay (see Methods S1 and Figure S1 for details). Using this assay, we were able to detect a statistically significant, age-dependent increase in oxidative mtDNA damage with age. Although only a moderate fit, the linear model (R squared value 0.39, slope is statistically different from zero (P = 0.029)) is more consistent with the data than any exponential model (Figure 4c). Moreover, total damage burden remained low with a maximum (at day 14) of 3.5 lesions per 10,000 base pairs, an approximately 84% increase compared to young animals (day 3). Finally, we employed a more detailed lipidomics analysis of different CL species to evaluate the age-dependent changes in this mitochondrial matrix lipid. Overall, we found that the highly polyunsaturated forms of CL declined more rapidly with age than relatively less unsaturated forms (Figure 4D). For instance, within the major CL species (CL 80:13–16), we found a significant decrease in highly unsaturated CL, with CL (20∶4) species declining by about 50% between day 3 and day 10 (P<0.005). By comparison, more saturated CL (20∶3) showed no significant decline within the major CL species (CL 80:13–16). Within the overall less abundant (CL 78:12–15) species, both CL (20∶4) and CL (20∶3) show marked decline with age, but this decline is again more pronounced for the less saturated CL (20∶4) species.

Together these data showed that there was a statistically significant and consistent increase in ROS production and associated molecular damage in ageing *C. elegans*. However, this increase, even in mitochondria, appeared quite small and linear (at most twice the levels of young worms) even at ages where there was obvious functional decline (Figure 1).

### The GA480 double mitochondrial SOD knockout mutant

A number of mitochondrial SOD mutants have been described in *C. elegans* [28]–[30]. One of the major results from these mutants, with respect to the mFRTA, was a surprising lack of reduction in lifespan in response to single and even multiple deletion of SOD genes [28]–[30]. However, another intriguing observation about these SOD mutants is consistent reports that suggest delayed development and reduced brood size [29], [30]. To gain further insight into possible reasons for these phenotypes, we applied our assays to a *sod-2/sod-3* double mutant (GA480), which lacks both forms of mitochondrial SOD.

**Figure 5:**
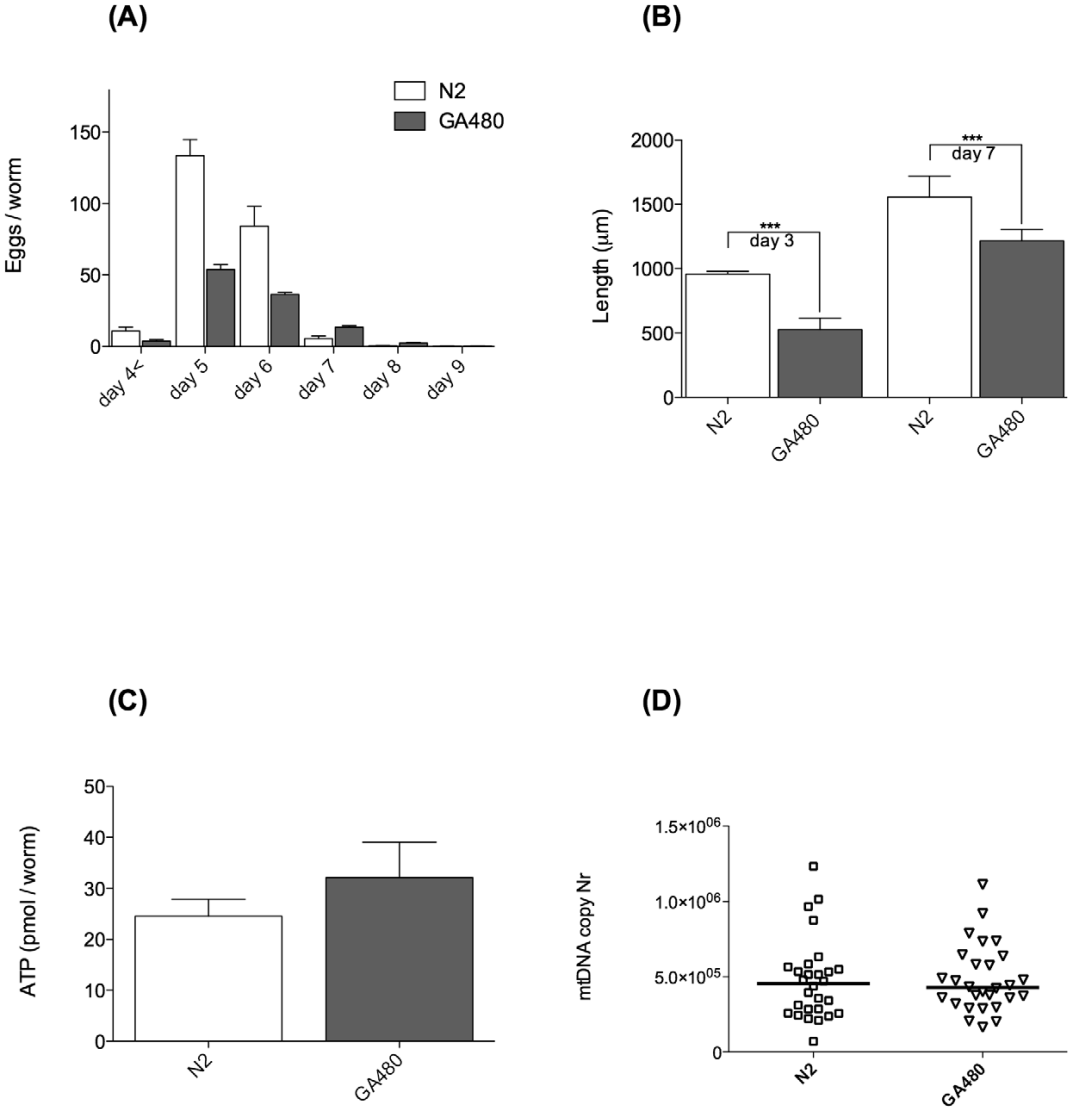
Phenotypical fitness comparison of GA480 *sod-2/sod-3* double mutant strain (*sod-2*(*gk257*) I, *sod-3*(*tm760*)) with WT N2 *C. elegans*. (**A**) Egg laying study comparing number of eggs laid daily by GA480 and WT worms (n = 10 each). GA480 produce significantly less eggs (total 222 vs. 107 eggs per adult on average, (P<0.0001, ANOVA with Tukey's post-test)). (**B**) GA480 worms grow slowly and are significantly smaller than WT on both day 3 and day 7 (both P<0.0001, Student's t-test). (**C**) Steady state levels of ATP per worm on day 4 are not significantly different between GA480 and WT worms (P = 0.18, Student's t-test). (**D**) No difference in mtDNA copy number in individual worms between GA480 and WT. Copy number was assessed on day 2 post bleach (n = 28 animals each), to minimise confounding effects of oocytes as much as possible. The median is not significantly different between GA480 and WT (P = 0.44, Mann-Whitney test).

**Figure 6:**
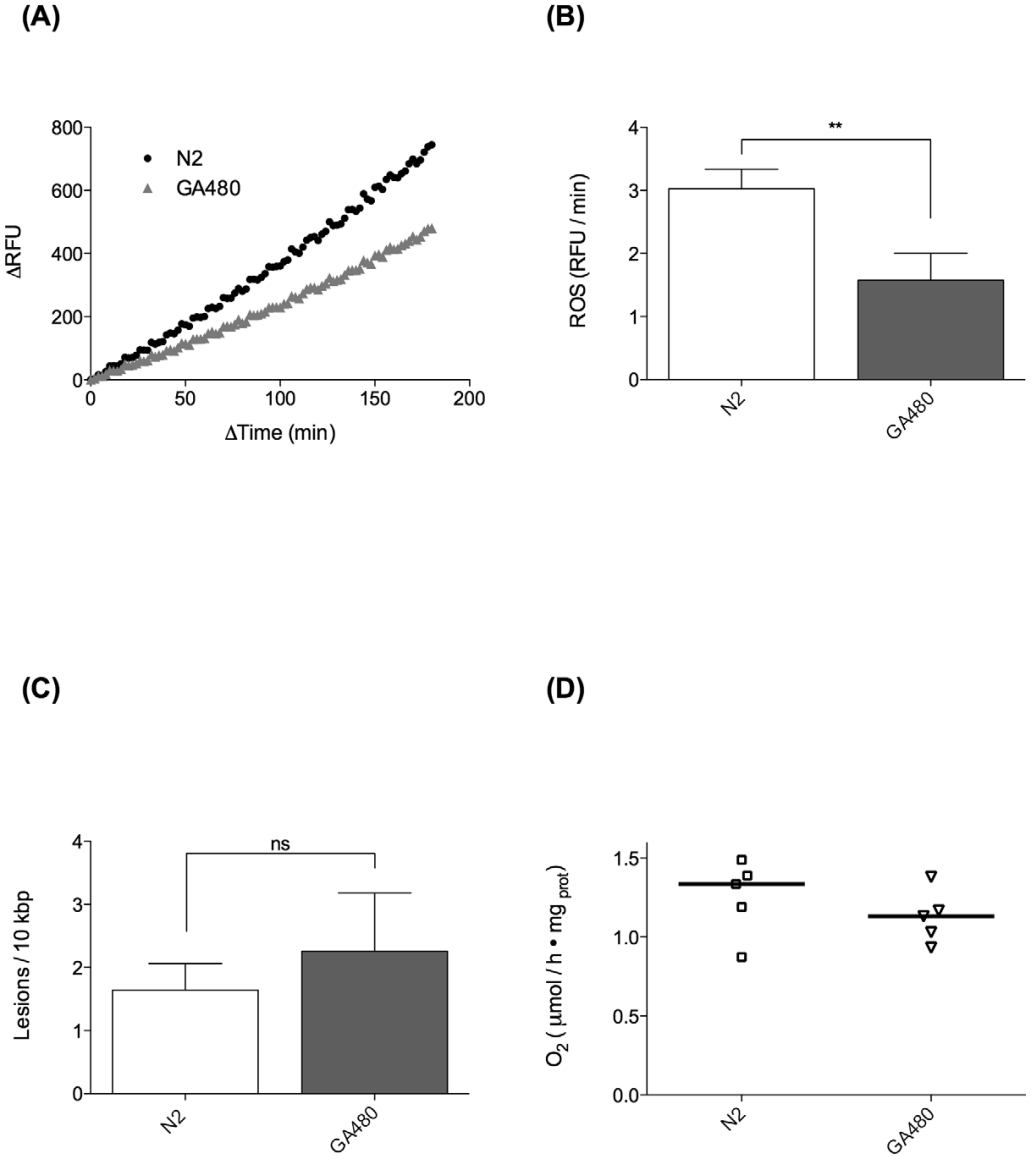
MnSOD mutants do not suffer from elevated oxidative damage. Comparison of ROS related parameters between MnSOD double mutant strain GA480 and WT N2 *C. elegans*. (**A**) Averaged fluorescence signal from whole worm DCF-DA generalised ROS assay (n = 7 repeats) of day 4 WT and GA480 animals. Despite lacking both forms of mitochondrial SOD, GA480 animals produce less ROS associated fluorescence signal than WT animals. (**B**) Analysis of the data from (A); GA480 produce statistically significantly less ROS as measured by our DCF-DA assay (P = 0.024, Student's t-test). (**C**) Oxidative mtDNA lesions in mtDNA samples (n = 4, each) of day 4 GA480 and WT worms. GA480 does not suffer from significantly elevated oxidative damage as judged by our qRT-PCR assay for oxidative mtDNA damage (P = 0.43, Student's t-test). (**D**) Oxygen consumption in GA480 vs. WT animals on day 4 of life. The median oxygen consumption was not significantly different between GA480 and WT (P = 0.31, Mann-Whitney test).

First, we characterised the extent of developmental and fecundity deficits in GA480 by determining the schedule of egg production, number of progeny on each day of adulthood and the adult size and growth rate of GA480 (Figure 5A and 5B). We found that GA480 produced approximately 50% less progeny than WT overall and also produced significantly fewer eggs on the peak days (days 5 and 6) of egg production (P<0.0001). Consistently, adult GA480 were also significantly smaller, with the length of GA480 being 55% and 78% that of WT on day 3 and day 7, respectively (Figure 5B). Together these data suggested to us that GA480 might suffer from a decrease in usable metabolic energy. To further investigate this possibility, we determined ATP levels, oxygen consumption and mtDNA copy number in age-matched GA480 and WT nematodes. While there was a trend towards slightly lower mtDNA copy number in GA480 relative to WT, neither this nor ATP levels were significantly different between mutant and WT (Figure 5C and 5D). While there was a trend towards reduction in oxygen consumption, this was not statistically significant (Figure 6D). Surprisingly, we found that ROS production, as determined by whole animal DCF-DA signal, was significantly *lower* in GA480 than in WT controls (Figure 6A and B). Furthermore, we found that steady-state mtDNA damage burden was *not* significantly higher in GA480 than in WT animals (Figure 6C). While there appeared to be a slight (non significant) trend towards elevated mtDNA damage burden, mtDNA damage was not elevated as dramatically in GA480 as might have been expected in a complete mitochondrial SOD knockout. In fact, mean mtDNA damage levels in 4-day-old GA480 were comparable to those in 8-day-old WT animals from our ageing cohort study and are certainly lower than the highest levels detected in our oldest animals (day 14).

### Making sense of SOD knockout mutants *in silico*

**Figure 7:**
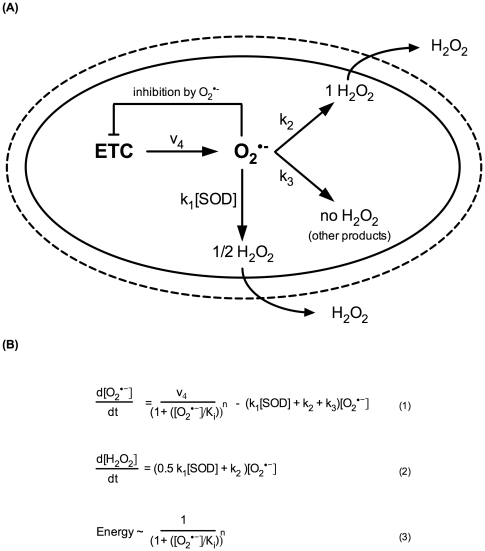
Minimal model for mitochondrial superoxide production and consumption and hydrogen peroxide production. Model based on that of Gardner *et al.* [37], [50], modified for the mitochondrial compartment. **A**) Three classes of superoxide consumption reactions with different hydrogen peroxide yields are considered. Each class represents a large number of actual reactions yielding either 1, 1/2 or no hydrogen peroxide per superoxide consumed. The model includes a simple negative feedback mechanism of superoxide on its own production rate. **B**) Equations for production and consumption of superoxide and hydrogen peroxide according to the model. Hydrogen peroxide, but not superoxide, can exit the mitochondria, and therefore, only equation (1) is steady state (d[O_2_•^−^]/dt = 0). The actual rate of superoxide production is v_4_/(1+[O_2_•^−^]/K_i_)^n^, where the denominator models the feedback inhibition of superoxide negatively modulating its own rate of production. The strength of this feedback is controlled by the exponent n, while the threshold at which feedback becomes significant is controlled by the inhibition constant K_i_. For n = 0, there is no feedback inhibition at any level of superoxide while, for n = 1, superoxide production is ½ maximal if superoxide levels are equal to K_i_.

In an attempt to explain the experimental results obtained for the GA480 SOD double mutant, we utilised a simple, analytically solvable model based on that of Gardner *et al.* [37], [50]. This model was originally designed as a minimal model to explain the paradoxical elevation of ROS-mediated damage sometimes observed when SOD is *overexpressed* (Figure 7, Table 1). While the model is deliberately minimal, it does reflect two of the most important mechanisms that have previously been shown to be relevant for the understanding of SOD modulation [35]–[37], [50].

**Table 1:** Model parameters.

Name	Value	Notes
v_4_	6.6×10^−7^ Ms^−1^	Rate of superoxide generation [35]
k_1_	2.3×10^9^ M^−1^s^−1^	Rate constant for MnSOD with superoxide [77]
k_2_	5×10^2^ s^−1^	Aggregate rate constant for reactions yielding a single molecule of hydrogen peroxide per superoxide consumed. The dominant processes considered are reactions between superoxide and susceptible dehydratases, including aconitase (cellular concentration up to 10^−4^ M [56], likely to be higher in mitochondria due to the mitochondrial localisation of, for instance, aconitase). Typical second order rate constants are 10^6^–10^7^ M^−1^s^−1^ ([11] p101). Reactions with ascorbate are also included here.
k_3_	1×10^3^ s^−1^	Aggregate rate constant for reactions not yielding hydrogen peroxide. The representative process considered is oxidation of ferricytochrome (mitochondrial concentration up to 5 mM [78], [79]) by superoxide. The rate constant for this process is 2.6×10^5^ M^−1^s^−1^. Reactions with NO are likely negligible due to low NO concentrations [80] and reactions with ubiquinol are thermodynamically unfavorable [50].
K_i_	7.8×10^−11^ M	Inhibition constant for feedback inhibition by superoxide. This was chosen to be identical to the level of superoxide calculated for the WT SOD case (SOD = 1) without feedback inhibition (n = 0). This choice for K_i_ means that at WT levels of SOD, “switching on” inhibition results in an approximately 40% inhibition (Inhibition constant = 0.6, see Figure 8). This is consistent with the significant increase (almost 50%) in ATP levels observed following the deletion of the uncoupling protein UCP4 in otherwise WT *C. elegans* larvae, as well as with estimates of approximately 25–30% baseline proton leak [52], [53].
[SOD]	3×10^−6^ M	WT concentration of MnSOD in mitochondria [77].
n	0 or 1	Order of feedback term: n = 0/no feedback inhibition, n = 1/linear feedback [50].

First, in addition to SOD-mediated dismutation, which yields one molecule of hydrogen peroxide per two superoxides, there are multiple SOD-independent pathways for superoxide consumption that can be classified by their different stoichiometry with respect to hydrogen peroxide production. These different pathways are modeled collectively as pseudo-first-order reactions with aggregate rate constants (Figure 7 and Table 1). Flux through these alternative pathways will change as levels of SOD are modulated and, under certain conditions, this can lead to counterintuitive results, for instance in terms of hydrogen peroxide production [35], [37]. Second, there are mechanisms by which high superoxide levels can inhibit mitochondrial function, thereby also indirectly modulating superoxide production rate [36], [51]–[55]. Here, this feedback inhibition of superoxide on its own production is modeled following the approach of Gardner *et al.* by equation 3 (Figure 7B). Parameter values used (Table 1) are based on those by [50] and [35] with modifications to adapt the model to the mitochondrial compartment. In particular, we assume here that, in the mitochondrial compartment, the reaction of superoxide with ferricytochrome likely contributes significantly to k_3_ while the major contributors to k_2_ are the 4Fe-4S clusters of aconitase and other susceptible dehydratases [11], [37], [56]. Fortunately, the qualitative behaviour of the model generally does not depend sensitively on the exact choice of these parameters.

### Modeling results

**Figure 8:**
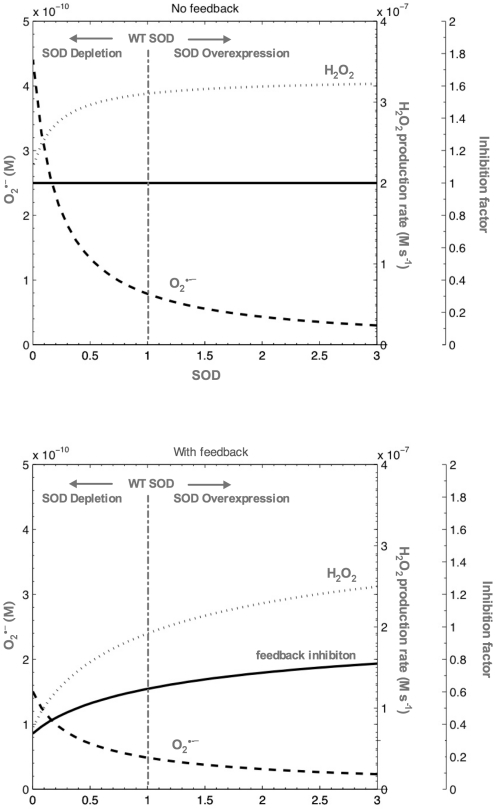
Results of SOD knockdown/deletion or overexpression according to the model. Model results showing dependency of mitochondrial superoxide level, hydrogen peroxide production rate and feedback inhibition factor (decrease in energy production relative to n = 0 case) on mitochondrial SOD without feedback (top, n = 0) and with feedback inhibition (bottom, n = 1). WT levels of mitochondrial SOD are normalised to 1 and indicated by the vertical dashed line. The area to the right of WT levels reflects SOD overexpression while the area to the left reflects SOD depletion, with complete knockout corresponding to zero SOD. Note that hydrogen peroxide production rate increases monotonically with SOD. This result is independent of the exact choice of parameters when feedback inhibition is considered (n = 1) as previously observed [50]. The inhibition factor on the third axis is a number between 1.0 and 0.0 that indicates the metabolic depression by feedback inhibition due to mitochondrial uncoupling and inhibition of ETC components by superoxide (relative to the n = 0 case). When the feedback inhibition factor is close to 1.0, metabolism and ROS production are maximal. In particular, when feedback is ignored (n = 0) the factor is 1.0 by definition. Note that, even at WT SOD levels, the feedback factor in the case of n = 1 is approximately 0.62. This can be interpreted as the effect of residual mitochondrial uncoupling, even at WT levels of SOD, which is consistent with the expected basal proton leak through UCP4 [53].

Solving the model under the assumptions outlined above revealed that superoxide does indeed increase when mitochondrial SOD decreases (Figure 8). However, due to superoxide consumption through the alternative pathways (via k_2_ and k_3_), this increase remaines well below one order of magnitude (5–6 fold with the parameters chosen here), even if feedback inhibition was ignored (n = 0) (Figure 8 top). When inhibition was considered (n = 1), the increase in superoxide was further attenuated, such that superoxide levels, even in complete knockout mutants without any other (for instance genetic) compensation, at most rose approximately 3 fold (Figure 8 bottom). Importantly, even in the most unrealistic case of complete knockout without compensation and ignoring feedback inhibition, absolute steady state superoxide levels remained low, never rising above 0.5 nM.

Hydrogen peroxide production rates, by contrast, were on the order of 10–100 nmolar s^−1^ and *decreased* when mitochondrial SOD content was reduced (Figure 8). This is a consequence of the negative feedback mechanism and a key feature of this model, explaining the paradoxical increase in oxidative stress in SOD overexpression experiments [37], [50]. For the mitochondrial SOD knockout mutants, we found that hydrogen peroxide production rates are predicted by our model with and without feedback inhibition to be 39% and 71% of WT, respectively.

With the parameters chosen here, the feedback inhibition factor (Ki) was 0.62 for SOD WT (SOD = 1) and 0.29 for complete SOD knockout mutants (SOD = 0) (Figure 8 bottom). Since this inhibition factor models processes such as mitochondrial uncoupling and/or inhibition of ETC components by superoxide, this can be interpreted as a drop in total usable metabolic energy flux by approximately 50% with respect to WT (from 0.62 to 0.29, where a factor of 1.0 corresponds to the theoretical maximum obtained without any feedback inhibition (n = 0)).

## Discussion

The first result from our WT ageing cohort study is that there is little support for the “vicious cycle” theory in *C. elegans*. The age-dependent oxidative damage accumulation and increase in ROS production are linear and limited (Figure 4). However, the notion that mitochondria and mitochondrial ROS do matter for ageing is supported by our results regarding the rapid age-dependent decline in markers of energy metabolism (Figure 2) as well as the better preservation of mtDNA copy number in more successfully ageing worms (Figure 3).

While ROS related parameters change only linearly with age, metabolic decline, especially post reproduction, is rapid in *C. elegans* (Figure 2) [19], [20], [44], [45]. One of the major challenges for the damage accumulation theories of ageing is to explain if and how a moderate, linear accumulation of damage with age can cause the rapid decline in systems performance and the exponential increases in morbidity and mortality seen during ageing [8], [57]. Given its role in mitochondrial function, the significant age-dependent change in content and composition of CL with age (Figure 2D and 4D) is a mechanism that warrants further investigation in this context.

Below, we contrast data from our ageing cohort studies with observations from the GA480 mutant strain to show a possible further link between moderate age-dependent increases in ROS and the significant decline in energy production with age. The lack of lifespan reduction in the mitochondrial SOD mutants has been interpreted as a major challenge to the mFRTA [12], [24], [28], [32], [33]. This interpretation is based on the assumption that complete knockout of mitochondrial SOD must be associated with a dramatic increase in oxidative damage, in particular to mitochondria, and that the lack of lifespan effects therefore indicates that ROS mediated damage to mitochondria does not determine the rate of ageing in *C. elegans*. Taken as a whole, our data reveal that the situation is more complicated. Although the mitochondrial SOD mutants do show increased susceptibility to artificially elevated oxidative stress [28]–[30], this does not prove that they suffer from dramatically elevated oxidative damage under normal conditions. Using our sequence-specific mtDNA damage assay, we failed to detect a significant increase in oxidative damage to mtDNA in the GA480 mitochondrial *sod-2/sod-3* double mutant. While our data do not rule out a slight increase in mtDNA oxidative damage, comparison with the ageing cohort study clearly shows that mtDNA oxidative damage levels in young SOD mutants are lower than those seen in old WT animals (Figure 4C and 6C).

The notion that oxidative damage is, at most, mildly increased in mitochondrial SOD mutants is also consistent with the limited biomarker data available in the literature. There is some evidence for a slight increase in the lipid oxidation product *trans*-4-hydroxy-2-nonenal in mitochondria of *sod-2* mutants [58]. In addition, the Hekimi lab reported a small (30%–40%) increase in oxidative protein damage in response to RNAi knockdown of *sod-1* and *sod-2* in WT *C. elegans* and an approximately 100% increase in protein carbonyl content in *sod-2* mutants [30], [31]. By comparison, we also detected an approximately 100% age-dependent increase in protein carbonyl content in our ageing cohort study and this magnitude is consistent with age-dependent increases in protein carbonyl content observed in *C. elegans* by others [19].

How is it possible that even complete loss of mitochondrial SOD does not cause oxidative damage more severe than indicated by these results? One hint towards a likely answer comes from reports that the SOD mutants are small, developmentally delayed and have reduced progeny. It is not immediately obvious why deletion of a class of antioxidant genes should result in such fitness penalties instead of increased mortality and reduced lifespan. However, one obvious way to overcome reduced ROS detoxification capacity is to produce less ROS in the first place [52], [59]. Mitochondrial uncoupling can modulate ROS production through the conserved uncoupling protein UCP4 in *C. elegans* and this can potentially result in a dramatic reduction of ROS production, although at significant cost in terms of energy production [14], [52], [53], [60], [61]. In this context, the reported increase in *trans*-4-hydroxy-2-nonenal in *sod-2* mutants is of interest, as this lipid oxidation product has previously been shown to activate mitochondrial uncoupling proteins [58], [62].

Similarly, there is evidence for preferential effects of oxidative modification on specific mitochondrial proteins, including aconitase, involved in energy production [54], [55]. Therefore, even a moderate increase in total protein carbonyls, as detected in the SOD mutants, could be associated with a significant depression of ETC function. This explanation is consistent with further evidence that treatment with N-acetyl-cysteine (NAC), presumably reducing mitochondrial ROS, leads to increased oxygen consumption and elevated ATP levels in both WT and some mitochondrial mutants [34]. This evidence supports the notion that mitochondrial superoxide is an important modulator of mitochondrial function, with even slightly elevated superoxide levels suppressing metabolism and extending lifespan and vice versa.

Using a simple mathematical model of these processes, we have shown how SOD knockout by this mechanism might result in significantly decreased metabolism and associated decrease in hydrogen peroxide production with only a moderate increase in mitochondrial superoxide levels. Given these results, it is perhaps not surprising that the effect of mitochondrial SOD knockout in nematodes is delayed development and decreased fecundity, rather than dramatically increased oxidative damage to mitochondria and shortened lifespan. The situation in higher organisms is likely complicated by the fact that they are less able to respond to reduced availability of metabolic energy, explaining the more severe phenotypes of mitochondrial SOD knockout in such species [63], [64]. Of course, hydrogen peroxide and superoxide are also likely to affect signaling, including through the insulin-like growth factor pathway, and changing their relative abundance might have signaling effects that contribute to the observed fitness effects as well as explaining some of the longevity effects [29].

Some additional insights into the plausibility of our model can be derived by considering ROS production in SOD mutant strains as measured by different methodologies. Using the whole worm DCF-DA dye assay, we found that DCF-DA associated fluorescence in whole animals was (∼50%) lower in GA480 than in WT. While there are questions regarding the localisation and identity of the chemical species measured by DCF-DA [65], [66], it is clear that DCF-DA does not directly detect superoxide [11]. We therefore interpret this result as general reduction of total ROS flux. Since hydrogen peroxide, but not superoxide, can exit mitochondria, the observed 50% drop in DCF-DA signal then is consistent with the predicted ∼50% decrease in metabolism and hydrogen peroxide production rate in SOD knockout mutants (Figure 8 bottom). Others, using the mitochondrial superoxide detector dye MitoSOX red [58], [67], have found a slight (at most 29%) increase in MitoSOX fluorescence in single mitochondrial SOD mutants [58]. The authors suggest that this increase is due to elevated mitochondrial superoxide content, but argue that the actual increase is likely to be somewhat higher (as much as two-fold). These *in vivo* data are also consistent with results in extracted mitochondria [68]. If these data are accepted, the experimental results together indicate a moderate increase in intramitochondrial superoxide levels but decrease in overall ROS flux in response to loss of mitochondrial SOD, a result entirely consistent with our model predictions (Figure 8 bottom).

This interpretation further suggests increased uncoupling, reduced mitochondrial membrane potential and/or reduced ETC activity as major consequences of mitochondrial SOD deletion. Specifically, the model predicts that the available metabolic energy in the mitochondrial knockout mutants should be ∼50% lower than in WT. This is broadly consistent with observations in terms of the magnitude of reduction in progeny and with the reduction in eggs laid on the peak egg-laying days (Figure 5A). Steady-state ATP levels, which were not different between GA480 compared to WT, are a function of both energy production and consumption rates and do not by themselves allow conclusions regarding ATP flux. In contrast to one report in the literature [30], we did not detect a significant decrease in oxygen consumption in GA480, although there was a trend to lower oxygen consumption (Figure 6D). However, given that both ETC inhibition and uncoupling are expected to contribute to feedback inhibition, our model does not easily allow prediction of oxygen consumption because partially uncoupling mitochondria would increase oxygen consumption while ETC inhibition would decrease it. In this context, it might be interesting to evaluate the effects of mitochondrial SOD mutation in a UCP4 knockout mutant background [53]. In summary, against expectation, we found that the mitochondrial SOD double mutant strain GA480 showed evidence for *reduced* ROS release and did *not* suffer from significantly elevated oxidative mtDNA damage. Instead, these mutants seem to experience a significant (up to 50%) decrease in available metabolic energy, as judged by growth and total, as well as peak fecundity.

Given that these results suggest that normal or even extended survival is possible in the presence of elevated mitochondrial superoxide levels, do the mitochondrial SOD mutants show that mitochondrial ROS do not matter for ageing in *C. elegans*? Not necessarily; our model suggests that keeping mitochondrial superoxide below a very low (sub nM) threshold is achieved by the mitochondrial regulatory system, even though doing so comes with significant penalties in terms of energy and ultimately fitness. That a conserved mechanism governing such a tradeoff appears to have evolved actually supports the notion that mitochondrial ROS are important determinants of survival and life history choices. It is interesting to note that the markers of oxidative damage and ROS production measured in SOD mutants by us and others remain below the levels seen in old *C. elegans*. It is possible, therefore, that oxidative damage both in ageing animals and in mutants remain low for the same reason, namely, that levels above a fixed, tightly controlled threshold will trigger ETC inhibition and mitochondrial uncoupling. In this scenario, the main purpose of mitochondrial SOD is not to limit global oxidative damage but to maintain mitochondrial function by preventing the superoxide “rheostat” [51] from engaging. Attempts at modulating mitochondrial ROS via the antioxidant system are then almost certain to predominantly modulate energy production rather than ROS mediated damage. This also makes any “vicious cycle” difficult to envisage because, once ROS levels cannot be kept below the threshold any longer in old animals, energy metabolism will collapse to the point where it is inconsistent with tissue function and organismal survival. Finally, the observation that modulation of mitochondrial function via mitochondrial superoxide is clearly closely linked to lifespan regulation raises the question by what mechanism reduction of ETC flux causes lifespan extension. The assays described here as well as complementary methods recently described by others [69], [70] open up exciting future opportunities for the elucidation of the possible role of mitochondria and mtDNA in this context.

## Materials and Methods

### Nematode strains and maintenance

The Bristol N2 (WT), JK1107 (*glp-1*) and GA480 (*sod-2/sod-3*) *C. elegans* strains were used. Worms were maintained at 20°C on nematode growth medium (NGM) agar plates with the exception that JK1107 worms were cultivated at 25.5°C to prevent progeny. Preparation of NGM was as previously described [71] with the addition of 200 µg/ml final concentration of Streptomycin. Streptomycin resistant *Escherichia coli* strain OP50-1 was added (50 µl of 10^10^ cells/ml *E. coli* stock per 35 mm petri dish and 500 µl of *E. coli* per 94 mm petri dish) to each plate. Worms used in the lipidomics study were grown in liquid culture [71] but synchronised on the same day as those grown on plates. This was necessary to obtain sufficient numbers for the lipid extraction protocol.

### Cohort studies

All age-dependent patterns reported were observed consistently in at least two independent ageing cohort studies. Assays for each worm population were performed at least in triplicate.

### Nematode size

Worms were photographed as part of the egg laying study using a calibrated Leica MZ10F microscope (Leica, Singapore). Worm size (in µm) was determined with the free curve tool provided by the Leica Application Suite software (v2.6.0 R1).

### Pharyngeal pumping

Videos of worms were recorded on days 4, 8, 12 and 14 at 80× magnification using a dissecting microscope (Leica MZ16) with attached camera (DFC 300 FX). At least 3 minutes of video were recorded for each animal (n = 10) and viewed in slow motion for accurate quantification.

### Motility phenotype

Age-synchronised worms were assessed both for spontaneous locomotion and for response to prodding with a platinum wormpick as described in [40].

### Lifespan studies

Worms were counted every 1 to 3 days of adulthood, depending on prevalent mortality. Those that failed to respond to mechanical touch were scored as dead and removed from the plates. Worms that had crawled off onto the sides of the plate and died away from the agar were censored.

### Egg laying assay

Mutants or N2 WT nematodes (n = 10, each) were transferred from hatching plates to individual egg-laying plates on day 1 of adulthood. Animals were thereafter transferred every 24 h to fresh egg-laying plates until egg laying ceased. Eggs laid were allowed to hatch at room temperature and progeny were counted after 2 days.

### ATP assay

The adenosine triphosphate (ATP) assay was performed as described in [41] with minor modifications. Briefly, flash frozen nematodes were re-suspended in trichloroacetic acid, lysed and incubated on ice, followed by centrifugation. Supernatants were pooled, and aliquots of ATP standards or sample supernatant were pipetted into a white 96-well plate, followed by addition of arsenite ATP buffer. ATP levels were measured using a luminometer (Infinite 200, Tecan, Männedorf, Switzerland) preprogrammed to inject firefly lantern extract.

### Oxygen consumption

The metabolic rate of whole *C. elegans* was determined using a Clark-type oxygen electrode (Hansatech, UK). Worms were washed, re-suspended in 1 ml M9 buffer (for 1 l: 3 g KH_2_PO_4_, 6 g Na_2_HPO_4_, 5 g NaCl, 1 ml MgSO_4_ [1 M]), and then placed in the electrode chamber, which had earlier been stabilised for 30 min with 1 ml air-saturated M9 buffer. Changes in oxygen concentration were monitored polarographically for 2–15 min to obtain oxygen consumption rates that were later corrected against bacterial controls. Final oxygen consumption rates were normalised by protein concentration using the D_c_-Protein Assay kit (Bio-Rad, Hercules, USA).

### Measurement of general reactive oxygen species (ROS)

100 worms were transferred into each well of a black 96-well plate containing 100 µl M9 buffer. Dichlorofluoresceine-diacetate (DCF-DA) (50 µM in M9 buffer, 100 µl, Invitrogen, Carlsbad, USA) was then added to each well. ROS associated fluorescence levels were measured kinetically using a fluorescence plate reader (SpectraMax Gemini EM, Molecular Devices, Sunnyvale, USA) at excitation 485 nm and emission 520 nm, room temperature, every 2 min for 14 h. Data were normalised to bacterial controls, composed of 100 µl OP50-1-containing M9 buffer in separate wells on the same plate.

### Protein carbonyl determination

Protein carbonyls were determined according to [41]. Briefly, worms were collected, washed, re-suspended in PBST (0.1% Tween-20 in PBS, 1st BASE, Singapore) containing 1 mM phenylmethylsulfonyl fluoride, and sonicated on ice. Protein concentrations of the lysates were determined by the D_c_-Protein Assay (Bio-Rad, Hercules, USA). Samples were derivatised, incubated at room temperature, and neutralisation solution added. Sample lysates were loaded into a slot blot apparatus (Bio-Rad, Hercules, USA) and then transferred onto a nitrocellulose membrane (Bio-Rad, Hercules, USA). After blocking for non-specific binding, the membrane was probed with anti-2,4-dinitrophenylhydrazine antibody (Chemicon International, Temecula, USA), followed by secondary detection with HRP conjugated anti-rabbit IgG antibody.

### Mitochondrial copy number quantification

Mitochondrial copy number was quantified according to [41]. Briefly, individual worms were picked into PCR tubes, lysed, and mtDNA copy number of individual nematodes was determined by quantitative real-time PCR (qRT-PCR,) using a reference sample of known copy number (quantified by serial dilution).

### Sequence-specific mtDNA damage

Sequence-specific burden of oxidative DNA lesions was determined based on digest of mtDNA with the formamidopyrimidine-DNA glycolase (fpg), followed by quantitative real time PCR (qRT-PCR) amplification. Fpg recognises and excises a range of modified DNA bases, including 8-oxoguanine, thereby rendering any templates containing at least one such lesion resistant to PCR amplification; the resulting drop in amplifiable template concentration can be converted into lesions per base pair (bp). Since sensitivity of this protocol depends on the length of the amplified template, a suitable long extension qRT-PCR protocol was developed based on [72]. See Methods S1 for more details on this protocol and data analysis.

### Analysis of lipids using high performance liquid chromatography/mass spectrometry (LC/MS)

Lipids were extracted using a modified Bligh and Dyer double chloroform extraction method [73]. Approximately 10,000 worms were homogenised in 0.9 ml ice-cold organic solvent mixture (chloroform: methanol, 1/2 v/v) and incubated on ice with agitation for 2 h in a vacuum container at a 4°C dark room. Subsequently, 0.3 ml of ice-cold chloroform and 0.45 ml of ice-cold water were added into the homogenate. The mixture was vortexed for 30 sec and centrifuged at 8600 g for 5 min, the lower organic layer was carefully transferred to a microcentifuge tube and a second extraction was then performed using 0.5 ml of ice-cold chloroform. The organic extracts were pooled, dried under a N_2_ stream and stored at −80°C until lipid analysis. Profiling of various lipids was carried out as described previously [74], [75]. Electrospray ionisation mass spectrometry (ESI/MS) was performed on a Waters Micromass Q-Tof micro mass spectrometer with an upfront Waters CapLC inlet (Waters Corp., Milford, MA). A Waters XTerra column (1 mm×150 mm) was utilised for separation of lipids. Chloroform∶methanol 1∶1 (v/v) with 5% of 300 mM piperidine (final 15 mM) was used as the mobile phase for isocratic elution. The mass spectrum was acquired from m/z 400–1650 with a frequency of 1 scan/s. An Agilent high performance liquid chromatography (HPLC) 1200 system coupled with an Applied Biosystem Triple Quadrupole/Ion Trap mass spectrometer (3200 Qtrap) was used for quantification of individual phospholipids and sphingomyelin species [75], [76]. Separation of individual classes of polar lipids was carried out using a Luna 3 µm silica column (i.d. 150 mm×2.0 mm). HPLC conditions were: mobile phase A (chloroform∶ methanol∶ ammonium hydroxide, 89.5∶10∶0.5), B (chloroform∶ methanol∶ ammonium hydroxide: water, 55∶39∶0.5∶5.5); flow rate 300 µl/min; 5% B for 3 min, then linearly changed to 30% B in 24 min and maintained for 5 min, and then linearly changed to 70% B in 5 min and maintained for 7 min. Then, the eluents were changed to the original ratio in 5 min and maintained for 6 min. Multiple reaction monitoring (MRM) transitions were set up for quantitative analysis of various polar lipids. Levels of individual lipids were quantified using spiked internal standards. PC-14∶0/14∶0, PE-14∶0/14∶0, CL-15∶0(3)/16∶1 and C12-SM standards were obtained from Avanti Polar Lipids (Alabaster, AL, USA).

### Statistical analysis

For statistical analysis GraphPad Prism version 4.00 for Apple Macintosh, GraphPad Software, San Diego (USA) was used. Data are reported and plotted as mean ± SEM unless otherwise stated. Group differences were assessed using unpaired t-test (single comparison) or ANOVA/linear regression (multiple comparisons/change with time) with the exception of individual worm mtDNA copy number, which failed normality test and, therefore, median differences were analysed using non-parametric test (Mann-Whitney test). Differences with P<0.05 were considered as statistically significant.

### Modeling

The mathematical model was implemented, solved (numerical integration, zero point determination) and plotted using the GNU Octave computer language (GNU Linux) for numerical computation (http://www.octave.org).

## Supporting Information

Figure S1 **Example qRT-PCR curves for young (left panel) and old (right panel) worms.** Template concentration post fpg digest are lower since oxidative lesions have been digested, converting lesions into sequence gaps. Fpg digested samples therefore have higher Cts than mock digested samples. The difference in Ct between fpg and mock digested samples is defined as ΔCt = Ct_mock_−Ct_digest_. Note that with this convention ΔCt<0, usually.
(TIF)

Methods S1 (DOC)
