# Supplementary figures and images for: Mitochondrial Changes in Ageing Caenorhabditis elegans – What Do We Learn from Superoxide Dismutase Knockouts?
Source: PLoS One. 2011 May 18;6(5):e19444. doi: 10.1371/journal.pone.0019444 (PMC3097207; doi:10.1371/journal.pone.0019444)

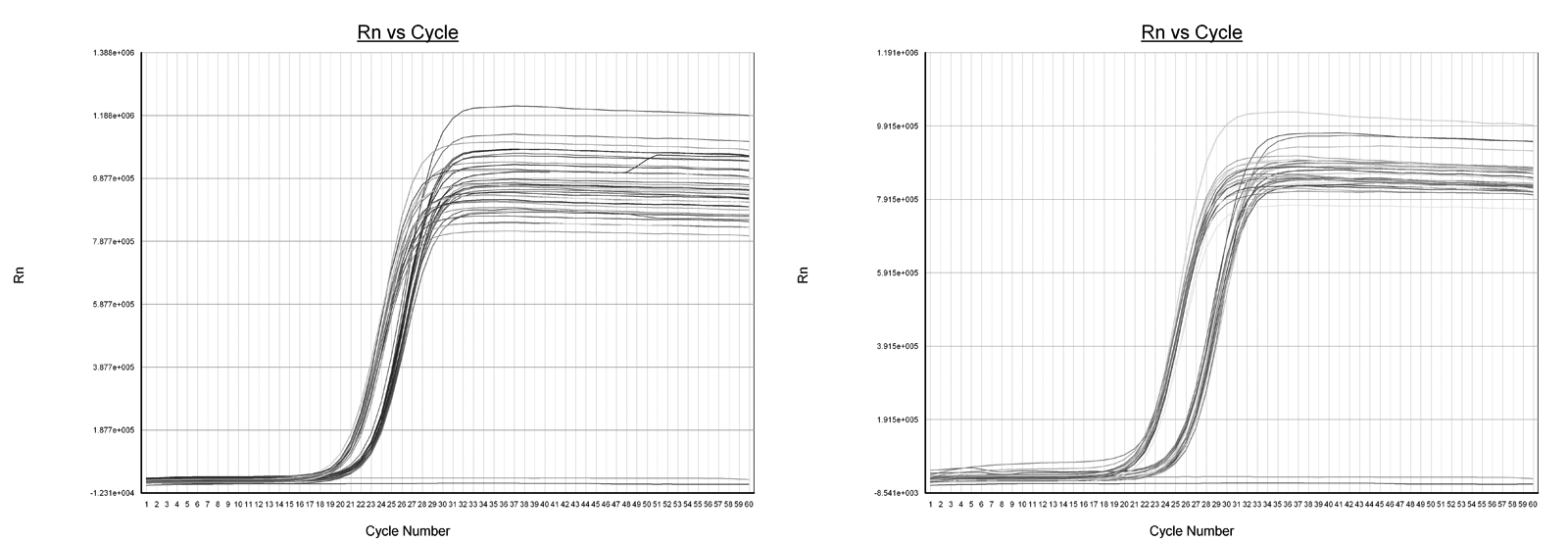

Supplement: Figure S1 — Example qRT-PCR curves for young (left panel) and old (right panel) worms. Template concentration post fpg digest are lower since oxidative lesions have been digested, converting lesions into sequence gaps. Fpg digested samples therefore have higher Cts than mock digested samples. The difference in Ct between fpg and mock digested samples is defined as ΔCt = Ctmock−Ctdigest. Note that with this convention ΔCt<0, usually. (TIF) [file pone.0019444.s001.tif]
