# Supplementary material for: Mitochondrial Changes in Ageing Caenorhabditis elegans – What Do We Learn from Superoxide Dismutase Knockouts?
Source: PLoS One. 2011 May 18;6(5):e19444. doi: 10.1371/journal.pone.0019444 (PMC3097207; doi:10.1371/journal.pone.0019444)
Supplement: Methods S1 — (DOC) [file pone.0019444.s002.doc]

**Methods S1**

**Sequence specific mtDNA damage assay**

The sequence specific burden of oxidative DNA lesions was determined based on the drop in PCR amplifiable template concentration (relative to mock digest) following digest of mtDNA with the formamidopyrimidine-DNA glycolase (fpg).

mtDNA preparation and Fpg digest:

Worms were washed off plates, allowed to settle on ice for 15 min, washed three times with ice-cold S-basal buffer (5.85 g NaCl, 1 g K_2_HPO_4_, 6 g KH_2_PO_4_, 1 ml 5 mg/ml cholesterol in ethanol, and dH_2_O to 1 l), and twice with ice-cold isolation buffer (210 mM mannitol, 70 mM sucrose, 0.1 mM EDTA, 5 mM Tris-HCl, pH 7.4) to remove dead animals. Removal of dead worms was confirmed visually under magnification. Worms were homogenised in isolation buffer in a total volume of 2 ml, on ice, using a Teflon-glass homogeniser (30 strokes at 1600 rpm). Worm homogenate was centrifuged at 600 g, 4 ^o^C, for 10 min; supernatant was then centrifuged at 7200 g, 4 ^o^C, for 10 min to pellet mitochondria, which were re-suspended in 100 μl TE buffer (50 mM Tris-HCl, 0.1 mM EDTA, pH 7.4). mtDNA was extracted using Prepman Ultra Sample Preparation Reagent (Applied Biosystems, Foster City, California, USA), according to the manufacturer’s protocol. For fpg digest, mtDNA (12 ng/μl, 20 μl) was incubated with fpg (0.1 U/μl, New England Biolabs, Ipswich, Massachusetts, USA) in 1 X NEBuffer 1 (New England Biolabs) and bovine serum albumin (BSA, 100 μg/ml, New England Biolabs) in a total volume of 40 μl at 37 ^o^C for 2 h, followed by “boosting” with additional fpg (0.1 U/μl in 1X NEBuffer 1, 4 μl) for another 2 h at 37 ^o^C. Fpg enzyme was then heat inactivated at 60 ^o^C for 10 min. A mock digest, omitting enzyme, was carried out in parallel for each sample. qRT-PCR was performed using the GeneAmp XL PCR kit (Applied Biosystems). Each 25 μl qRT-PCR reaction contained (final concentrations in brackets): 4.1 μl nuclease-free H_2_O (Promega, Fitchburg, Wisconsin, USA), 7.5 μl 3.3 X XL Buffer II (1 X), 2 μl GeneAmp 10 mM dNTP Blend (0.8 mM), 1 μl of each primer (0.4 μM, 1^st^ BASE, Singapore), 0.4 μl 25 mM Mg(OAc)_2_ Solution (0.4 mM), 2.5 μl BSA (100 μg/ml, New England Biolabs), 4 μl r*Tth* XL DNA polymerase (0.04 U/μl), 0.5 μl SYBR Green I nucleic acid gel stain (0.2 X, Molecular Probes, Eugene, Oregon, USA), and 2 μl sample (fpg or mock digested mtDNA) or nuclease-free H_2_O (No-Template-Control).

Primer sequences for mtDNA were based on [1]:

Forward (5’-3’) TCG CTT TTA TTA CTC TAT ATG AGC G

(nucleotides 1818-1842 mtDNA)

Reverse (5’-3’) TCA GTT ACC AAA ACC ACC GAT T

(nucleotides 8111-8090 mtDNA)

qRT-PCR was performed in MicroAmp optical 96-well reaction plates (Applied Biosystems) on an ABI 7500 Real-time PCR System (Applied Biosystems). The qRT-PCR cycling profile was an initial denaturation at 94 ^o^C for 2 min, followed by 60 cycles at 93 ^o^C for 10 sec, 62 ^o^C for 10 min, and 68 ^o^C for 30 sec, ending with a final extension at 68 ^o^C for 30 min.

Fpg recognises and excises a range of modified DNA bases, including 8-oxoguanine, thereby rendering any templates containing at least one such lesion resistant to PCR amplification (Figure S1).

Conversion of apparent drop in template concentration into lesions per bp

The observed drop in amplifiable template concentration (Figure S1) can be converted into lesions per base pair (bp) as follows:

To quantify oxidative DNA damage based on ΔCt we first calculated the ratio (R) of intact to cut PCR template in the fpg digest by comparing the observed Ct of digest versus mock digest:

R = k^ΔCt

The amplification factor (k) is a measure of the increase in PCR product per cycle. Under ideal conditions, fragment concentration would double in each PCR cycle (k=2). However, this is not necessarily the case, especially for long extension PCR. We therefore determined k by serial dilution of undigested DNA template (with our system: k = 1.68). Note that the value of k may differ for different experimental setups and DNA fragments.

If the target DNA has on average L ∈ [0,1] lesions per bp, the probability (P) of *zero* lesions in a given DNA fragment containing n bp can be easily calculated:

P = (1 – L)^n^

This probability of *zero* lesions in a given DNA fragment (P) is the same as the ratio (R) of intact (uncut) template to cut products in the fpg digest sample (see above):

=> P = R = k^ΔCt = 1.68^(Ctmock – Ctdigest)^

Using this relation, the number of fpg sensitive lesions per bp (L) can be simply calculated from the observed drop in template concentration following fpg digest:

(1 – L)^n^ = k^ΔCt = 1.68^(Ctmock – Ctdigest)^

=> L = 1 – 1.68^(Ctmock – Ctdigest) / n^

Note that the sensitivity of this method is thus dependent on the fragment length (n). Given the low levels of oxidative damage commonly found in DNA, this method therefore requires qRT-PCR of long fragments, in our case utilising a 6.3 kb fragment of mtDNA [1].

1. Melov S, Lithgow GJ, Fischer DR, Tedesco PM, Johnson TE (1995) Increased frequency of deletions in the mitochondrial genome with age of Caenorhabditis elegans. Nucleic Acids Res 23: 1419-1425.
